# Public perception of female paramedics at King Abdulaziz Medical City, Saudi Arabia

**DOI:** 10.1186/s12245-018-0217-4

**Published:** 2018-12-20

**Authors:** Nesrin Alharthy, Sara Alswaes, Alanoud Almaziad, Nourah Alenazi, Maha Abdallah, Moeed Alshehry

**Affiliations:** 1 College of Applied Medical Sciences, King Saud bin Abdulaziz University for Health Sciences, P.O. Box 22490, Riyadh, 11426 Saudi Arabia; 2 Pediatrics Emergency Department, King Abdulaziz Medical City, Riyadh, Saudi Arabia; 3 Emergency Medical Services Program, College of Applied Medical Sciences, King Saud bin Abdulaziz University for Health Sciences, Riyadh, Saudi Arabia

**Keywords:** Emergency medical services, Female paramedic, Public perception, Conservative culture

## Abstract

**Background:**

Although emergency medical service (EMS) providers recognize that both male and female paramedics are necessary, Saudi EMSs are currently fully staffed by men. Cultural bias against care provision by male paramedics to female victims in the absence of male guardians underscores the need for female paramedics. Consequently, we explored public perception of female paramedics at King Abdulaziz Medical City (KAMC), Riyadh.

**Method:**

This observational, cross-sectional study used convenience sampling to assess the perceptions of patients, visitors, and employees at the emergency rooms in KAMC and King Abdullah Specialized Children’s Hospital via self-administered English- and Arabic-language questionnaires. Questionnaire reliability and validity were assessed in a pilot study.

**Results:**

Three hundred twelve respondents completed the survey (67.30% men). The sample included 43.27% medical (40% paramedics, 22% physicians, 12% nurses, and 23% other) and 56.73% nonmedical participants, of whom 53% and 63%, respectively, strongly agreed regarding the importance of female paramedics. Moreover, in the male participant group, 6% of medical and 8% of nonmedical participants strongly disagreed with treatment of their female relatives by male paramedics, and 20% of medical and 30% of nonmedical participants declined medical help because female paramedics were unavailable.

**Conclusions:**

Respondents rated the importance of trained female paramedics in the EMS system. Most strongly agreed that female and male paramedics had equal patient-management capabilities and skills.

## Background

An emergency medical service (EMS) is defined as a comprehensive system involving a network of personnel, equipment, and resources established mainly to deliver emergency aid and medical care to the community [1]. In many cases, the lives of sick and injured patients depend on the rapid responses and competency of EMS professionals [2]. The EMS system mainly comprises a dispatcher, emergency medical technician (EMT), paramedic, and medical director. EMTs possess basic skills (e.g., cardiopulmonary resuscitation [CPR]), implement interventions (e.g., administration of oxygen), and treat allergic reactions or asthma attacks. Paramedics are highly educated and have more advanced skillsets, which include the administration of medications, placement of intravenous lines, and provision of advanced airway management [1]. In addition, paramedics can also perform complete physical examinations.

EMS providers recognize that the position of paramedic is not restricted to men. Accordingly, women must be encouraged to specialize in this field. During the World War II, women replaced male firefighters who served in the military. In the 1950s, the first basic EMT training program in the USA was implemented by the Chicago Fire Department [3]. By the 1970s, women had become more common in the ranks of regular volunteer fire departments [4] and have since demonstrated their ability to participate in the EMS field. In 1978, three female paramedics who had graduated were employed by the Los Angeles Fire Department [5].

Patients have the right to either accept or refuse the care offered by paramedics. A 2011 study of the effects of sex on patients’ refusal of prehospital care showed that the frequency of refusal decreased when paramedics were women [6]. The need for female paramedics is increasing internationally, particularly in conservative cultures such as the Kingdom of Saudi Arabia (KSA), which is known to hold the utmost respect for traditional customs. In 2015, Hamma and colleagues reported that up to 17.7% of patients in Saudi society would oppose the provision of care by a male paramedic to a female patient in the absence of a male guardian [7]. In addition, sex differences have led to several complications. For example female student of one university in Riyadh, who had known heart problems, collapsed after experiencing a heart attack and eventually died because the male paramedic could not access the patient on timely fashion [8].

Arab Gulf countries have recently begun to address this problem. For example, in 2010, various locations in Kuwait allowed female paramedics to work night shifts [9]. In the KSA, Dr. Lubna Al-Ansari, a Saudi consultative council member, described the need for female paramedics in the Saudi Arabian community to Al-Eqtisadiah Daily. She suggested that women should be trained and employed because “the presence of female paramedics will remove the embarrassment many women face when dealing with male paramedics” [10]. Female paramedics are urgently needed in the KSA, which is one of the most religious and conservative countries and implements strict rules regarding interaction between men and women. The KSA recently began training female paramedics in private colleges, and in 2015, King Saud bin Abdulaziz University for Health Sciences, a public university, began an EMS training program for women.

In the present study, a survey was conducted to measure public perception of female paramedics in King Abdulaziz Medical City (KAMC), Riyadh.

## Methods

This observational, cross-sectional study was conducted in the emergency department of KAMC, at which 300–450 patients are treated daily, and the pediatric emergency department of King Abdullah Specialist Children’s Hospital (KASCH), at which 260–400 patients are treated daily. Furthermore, the study sample included EMS providers from the National Guard Health Affairs. As per institutional review board approval, a convenience sample included participants from the waiting areas of the KAMC and KASCH emergency care center, Saudi health professionals employed at the KAMC and KASCH, and Saudi EMS personnel. Patients transferred from the emergency care center resuscitation unit at KAMC, pediatric patients, illiterate individuals, and non-Saudi individuals were excluded from the study. Moreover, informed consent was obtained from all respondents prior to data collection.

The current literature was deficient; therefore, we devised a self-administered questionnaire in both English and Arabic to identify the public perception of female paramedics in the KSA. A pilot study of 15 paramedic students and emergency nurses was conducted to test and validate question clarity and logic. To ensure reliability, a 5-point Likert scale was used to score the responses.

### Statistical analyses

Data were reported as frequencies and percentages for categorical data, and descriptive statistics were used. Percentages represented levels of agreement assessed using the 5-point Likert scale. The Mann-Whitney *U* test was used to compare the percentages of medical and nonmedical participants who agreed and disagreed with the employment of female paramedics. The effect size was declared as significant if *p* < .05. Cronbach’s *α* was used to assess the internal consistency and reliability of the questionnaire. SPSS version 20 (SPSS, Inc., Chicago, IL, USA) was used to perform statistical analyses.

The study design was in accordance with the principles of the Helsinki Declaration and approved by the research board at King Abdullah International Medical Research Center.

## Results

Three hundred twelve individuals (67.30% men; response rate = 80%) participated in the study. Cronbach’s *α* for the internal consistency of the questionnaire was .676, indicating moderate reliability. Participants’ demographic characteristics are shown in Table 1. The sample included 43.27% medical (40% paramedics, 22% physicians, 12% nurses, and 23% other) and 56.73% nonmedical participants.

**Table 1:** Participants’ demographic characteristics and medical personnel’s responses regarding work experience with female paramedics

Variable	Frequency			Percentage		
	Medical	Non-medical	Total			
Age (years)						
18–20	6	8	14	4.49		
21–30	83	83	166	53.20		
31–40	40	60	100	32.05		
41–50	6	20	26	8.33		
> 51	0	6	6	1.92		
Total	135	177	312	100		
Sex						
Male	97	113	210	67.30		
Female	38	64	102	32.69		
Total	135	177	312	100		
Level of education						
Primary	0	7	7	2.24		
Secondary	0	15	15	4.80		
High school	15	76	91	29.16		
Bachelor’s degree	83	65	148	47.43		
Master’s degree	8	1	9	2.88		
PhD	8	2	10	3.20		
Other	21	11	32	10.25		
Total	135	177	312	100		
Current status						
Employed	101	105	206	66.02		
Self-employed	0	9	9	2.88		
Looking for work	0	30	30	9.61		
A student	34	18	52	16.66		
Retired	0	14	14	4.48		
Total	135	177	312	100		
Percentage	43.27%	56.73%	100%			
The response of the medical personnel of work experience with female paramedics						
Question	Strongly agree	Agree	Undecided	Disagree	Strongly disagree	Total
Are female paramedics skilled enough to perform their work?	51 (63.75%)	19 (23.75%)	9 (11.25%)	1 (1.25%)	0 (0%)	80 (59.26%)
Your level of agreement regarding working with female paramedics?	38 (47.5%)	33 (41.25%)	8 (10%)	0 (0%)	1 (1.25%)	80 (59.26%)

**Fig. 1:**
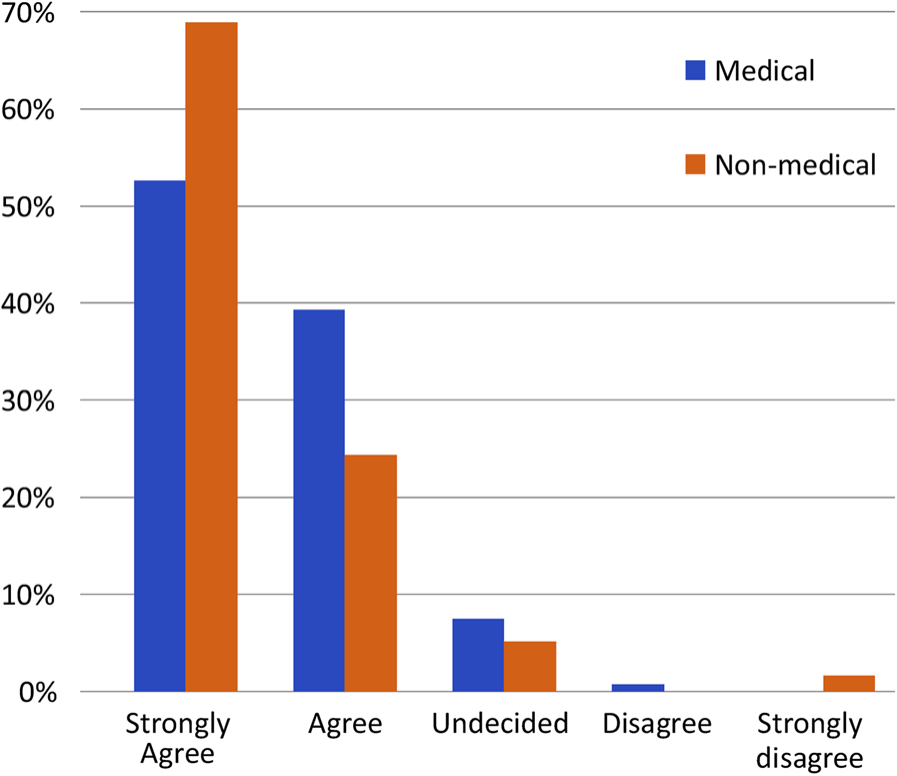
Level of agreement that employing female paramedics in Saudi Arabia is important

**Fig. 2:**
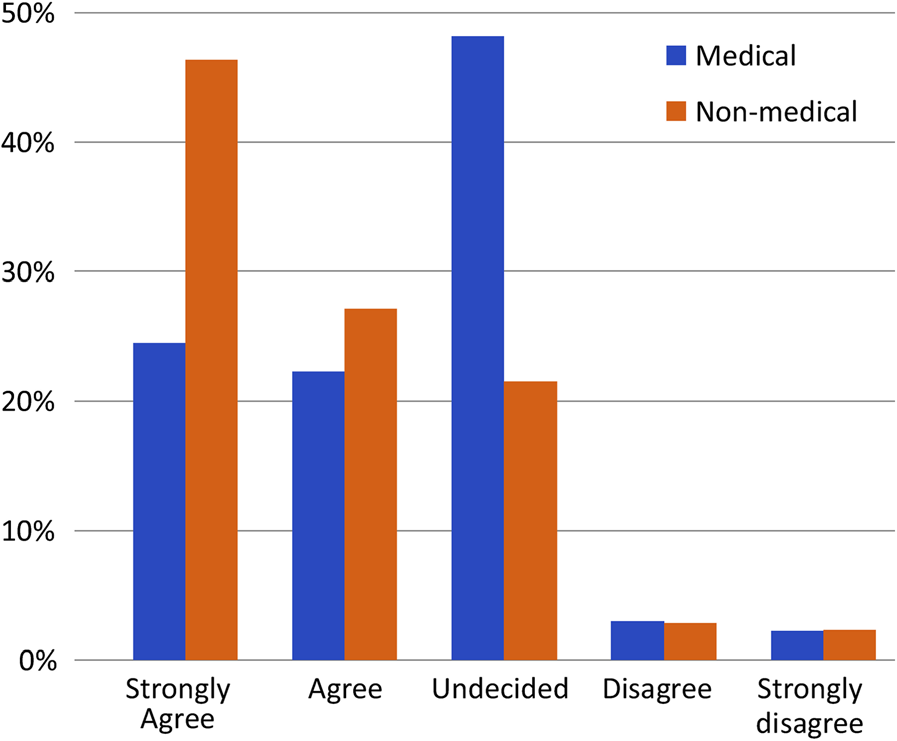
Preference for care provided by female paramedics in Saudi Arabia

**Fig. 3:**
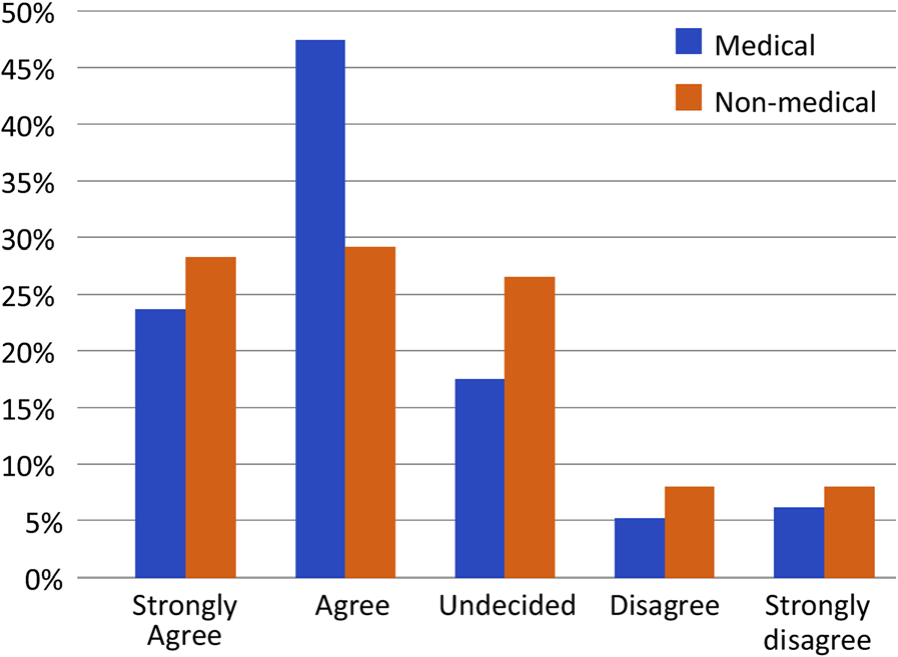
Male participants’ agreement with care of female relatives by male paramedics

The majority of both medical (63%) and nonmedical participants (53%) strongly agreed that employing female paramedics was important (Fig. 1). Of the latter, 46.33% strongly agreed that they preferred to receive treatment from female paramedics, 21% were undecided, and 2.26% strongly disagreed. In contrast, 24.44% of medical employees reported a strong preference for treatment from female paramedics, 48% were undecided, and 2.22% strongly disagreed (Fig. 2). Most participants who required the EMS did not refuse care because of a lack of female paramedics; however, 20% of medical employees and ≥ 30% of nonmedical participants reported that they had previously avoided contacting the EMS because of the lack of female paramedics. In the male participant group, 6.19% of medical personnel and 7.96% of nonmedical participants felt that male paramedics should not provide care to their wives, mothers, or sisters; however, 30–40% felt that male paramedics could do so, and 25–30% were undecided (Fig. 3).

In the analysis, a significant level of agreement regarding the presence of female paramedics was observed between nonmedical and medical participants; further, there was no significant difference between other medical participants and paramedics (Table 2).

**Table 2:** Comparison of mean scores between medical and nonmedical participants/other medical respondents and paramedic

Group	Mean **±** standard deviation	Test used	Test statistic	*P* value
Medical	1.563 ± 0.664	Mann-Whitney test	*U* = 10,189	.009
Nonmedical	1.435 ± 0.788			
Other medical	1.513 ± 0.595	Mann-Whitney test	*U* = 2060	.482
Paramedic	1.636 ± 0.754			

## Discussion

EMSs in the KSA currently employ only male paramedics. However, female patients could experience delays in prehospital care because of the conservative culture, which heightens the need for trained female paramedics. Recently, a few universities have begun to offer structured EMS training to female students. For example, King Saud bin Abdulaziz University for Health Science has introduced an EMS bachelor’s degree program for women, which is among the first structured curricula in this field at a public university. This EMS program aims to provide well-trained female paramedics to serve the region.

Despite these new programs, public knowledge and perception of female paramedics had not been explored previously in Saudi Arabia. Therefore, given its focus on public perception of female paramedics, our study is unique. Our findings indicated that most participants realized the importance of employing female paramedics in the KSA. Further, public concern regarding the preservation of women’s privacy and the cultural and religious beliefs common in the KSA were reflected in levels of agreement, in that 20 to 30% of participants had refused EMS contact in the past because no female paramedics were available.

Regarding sex preferences, female participants expressed strong agreement with accepting care from female paramedics, particularly in critical situations in which the presence of a male paramedic would be inconvenient. The existing literature also describes the refusal of hospital and other care because of sex preferences [6, 7]. Male participants indicated strong disagreement with the provision of care to their wives, mothers, and sisters by male paramedics, and they strongly agreed that male paramedics should be accompanied by female paramedics to overcome these sex limitations when treating female patients.

In the KSA, public awareness of this issue has increased following several media reports wherein female patients experienced serious complications because of cultural limitations that delayed the entry of male paramedics into women-only areas [11]. Most respondents reported that female paramedics could easily gain access to women-only areas and approach female patients in public places. In addition, most participants agreed that female paramedics were capable of handling very sick patients, regardless of sex or age. The perceived skills and patient management abilities of female paramedics in a prehospital setting were examined in a previous study, which showed a lower rate of prehospital care refusal among patients treated by female paramedics, relative to those treated by their male counterparts [6].

Moreover, most participants supported the pursuit of a paramedic career by women, considering cultural limitations. Our questionnaire included a suggestion box, in which participants could suggest limitations such as restricting the roles of female paramedic to women-only areas such as schools, colleges, and women’s health centers. Other participants highlighted the professional dress code and suggested that it should be modest, in accordance with the Islamic religion and traditions of the KSA. Further, participants listed some factors that could affect women’s ability, such as physical fitness, emotional liability, and the ability to work within an uncontrolled crowd. These suggestions indicated that the public is aware of the occupational hazards, particularly those faced by female paramedics, in accordance with previous studies that identified sex as a risk factor for post-traumatic stress disorder and occupational violence [12, 13].

A local review of the evolution of EMSs in the KSA showed that, despite advances in recent years, considerable opportunities remain for further improvement, particularly through increased public awareness and enhanced paramedic education [14]. Our study is unique in that we used a survey to assess public knowledge of and agreement with the existence of female paramedics, as this issue contributes to public concerns regarding sex preferences. Despite the paucity of existing literature, we examined public perception of female paramedics. The Saudi government aims to increase the number of women in the national workforce, which could involve staffing EMSs with female paramedics following increased demand and educational advancements [15]. However, the generalizability of our findings was limited by the sample size and study setting. Therefore, we recommend a national-level assessment of public perception of female paramedics.

## Conclusions

In conclusion, efforts should be made to empower female paramedics and meet the needs of the public. These efforts should target an increase in public knowledge and assess future challenges faced by female paramedics.
